# Identification of Differentially Expressed Genes in Leaf of *Reaumuria soongorica* under PEG-Induced Drought Stress by Digital Gene Expression Profiling

**DOI:** 10.1371/journal.pone.0094277

**Published:** 2014-04-15

**Authors:** Yubing Liu, Meiling Liu, Xinrong Li, Bo Cao, Xiaofei Ma

**Affiliations:** 1 Shapotou Desert Research & Experiment Station, Cold and Arid Regions Environmental and Engineering Research Institute, Chinese Academy of Sciences, Lanzhou, P. R. China; 2 Key Laboratory of Stress Physiology and Ecology in Cold and Arid Regions of Gansu Province, Cold and Arid Regions Environmental and Engineering Research Institute, Chinese Academy of Sciences, Lanzhou, P. R. China; 3 University of Chinese Academy of Sciences, Beijing, China; University of North Carolina at Charlotte, United States of America

## Abstract

*Reaumuria soongorica* (Pall.) Maxim., a resurrection semi-shrub, is a typical constructive and dominant species in desert ecosystems in northwestern China. However, the gene expression characteristics of *R. soongorica* under drought stress have not been elucidated. Digital gene expression analysis was performed using Illumina technique to investigate differentially expressed genes (DEGs) between control and PEG-treated samples of *R. soongorica*. A total of 212,338 and 211,052 distinct tags were detected in the control and PEG-treated libraries, respectively. A total of 1,325 genes were identified as DEGs, 379 (28.6%) of which were up-regulated and 946 (71.4%) were down-regulated in response to drought stress. Functional annotation analysis identified numerous drought-inducible genes with various functions in response to drought stress. A number of regulatory proteins, functional proteins, and proteins induced by other stress factors in *R. soongorica* were identified. Alteration in the regulatory proteins (transcription factors and protein kinase) may be involved in signal transduction. Functional proteins, including flavonoid biosynthetic proteins, late embryogenesis abundant (LEA) proteins, small heat shock proteins (sHSP), and aquaporin and proline transporter may play protective roles in response to drought stress. Flavonoids, LEA proteins and sHSP function as reactive oxygen species scavenger or molecular chaperone. Aquaporin and proline transporters regulate the distribution of water and proline throughout the whole plant. The tolerance ability of *R. soongorica* may be gained through effective signal transduction and enhanced protection of functional proteins to reestablish cellular homeostasis. DEGs obtained in this study may provide useful insights to help further understand the drought-tolerant mechanism of *R. soongorica*.

## Introduction

Water deficit is one of the most significant abiotic stresses that influence germination, growth, development, and productivity of plants [1]. Drought stress causes stomatal closure, limited gas exchange, and reduced photosynthesis in plants [2]. Over-reduction of photosynthetic electron transport chain induces the generation of reactive oxygen species (ROS), such as singlet oxygen (^1^O_2_), superoxide anion (O_2_^−^), hydrogen peroxide (H_2_O_2_), and hydroxyl radical (•OH), which damage cellular structures and macromolecules [3]. Plants have enzymatic and non-enzymatic systems to eliminate ROS. Moreover, plants have developed drought-resistance strategies such as succulent leaves, formation of osmophilic globules, stomatal movement, and reduction of leaf water potential [4], [5]. The understanding of plant responses to water deficit have improved because of the application of molecular techniques. The expression of numerous genes in response to drought stress was described in previous studies [6], [7]. Seki et al. [8] classified the genes expressed during stress into two groups: (i) genes encoding proteins involved in signal transduction (protein kinases and transcription factors) and (ii) genes with products, such as late embryogenesis abundant (LEA) proteins, chaperone, osmoprotectants, and detoxification enzymes, that directly protect cells against stress. Genomic and transcriptomic analyses revealed that various transcriptional regulatory systems are involved in stress-responsive gene induction. Several different sets of *cis*- and *trans*-acting factors are known to be induced by drought stress at the molecular level [9]. A large number of metabolites and proteins have also been reported to be up regulated in response to drought stress [10].

*Reaumuria soongorica* (Pall.) Maxim. is an extreme xerophytic semi-shrub and a typical constructive and dominant species in the desert vegetation in China. *R. soongorica* forms the zonal landscape and is widely distributed in northwest China [11]. Water supply is one of the main limiting factors in the habitat of this species. The unique adaptive strategies in the morphology and physiology of *R. soongorica*, such as thick cuticle, hollow stomata, and accumulation of some low-molecular-weight metabolites, are attributed to its special distribution area [4]. *R. soongorica* is a vascular flowering plant with desiccation-tolerance. *R. soongorica* leaves wither and enter a state of dormancy during dehydration but is revived when water becomes available. Thus, *R. soongorica* is utilized as a valuable non-model species in exploring drought-tolerance mechanisms. Liu et al. [4] found sucrose, malate, and proline, which play important roles in osmoregulation in *R. soongorica* during water loss. Studies on the protection mechanism for photosynthetic properties, activity of antioxidant enzyme, and metabolite changes under drought stress have also been conducted [12], [13]. However, the molecular mechanism of drought tolerance in *R. soongorica* is still poorly understood. A preliminary genome survey, which included genome size, chromosome number, and karyotype, was conducted with *R. soongorica* [14]. In our previous study, the mitogen-activated protein kinase gene (*RsMPK2*) was isolated and its participation as possible mediator under different stresses was investigated [15]. We also identified and characterized the response of flavonone 3-hydrolase gene (*RsF3H*), a key enzyme-encoding gene involved in flavonoid biosynthesis pathway in drought treatment [16]. Recently, the transcriptome of *R. soongorica* was sequenced and analyzed to obtain valuable genetic information for the investigation on the molecular mechanism of drought tolerance [17]. Genetic information from the transcriptome of *R. soongorica* was also used to identify differentially expressed genes (DEGs) under drought stress to understand the mechanism of drought tolerance.

In recent years, with the increasing availability of sequence data, expression profiling has been used to identify DEGs in different tissues, organs, developmental stages or mutants and determine the expression patterns induced by stress in a large number of model and non-model organisms. Microarray analysis and suppression subtractive hybridization are powerful tools for isolating differentially expressed cDNAs that mediate complex biological processes in higher eukaryotes [18]. These technologies have been used in a number of animals and plants, such as *Aedesaegypti* [19], soybean [20], *Populuscanadensis* [21], *Scylla paramamosain* [22], cucumber [23], and navel orange [24]. Next-generation sequencing technologies offer new approaches for global measurements of gene expression with the advantage of high efficiency and low cost. Digital gene expression (DGE) tag profiling is based on ultra-high-throughput sequencing of cDNA fragments that uniquely tag the corresponding gene, thereby allowing direct quantification of transcript abundance [25]. DGE technology allows identification of millions of DEGs without the need for prior annotations and permits the analysis of organisms that lack genomic information. Thus, DGE has been widely utilized to monitor the differences in transcriptional responses among different tissues and organs in response to stress in silkworm [26], *Sagittariatrifolia* [27], *Dugesia japonica* [28], *Populus* [29], cotton [30], spruce [31], *Brassica napus* [32], and moss [33].

In this study, DEGs in leaves of *R. soongorica* in response to PEG-induced drought stress were examined using DGE tag profiling technology. Analysis of gene expression related to stress response provides further insight into the molecular mechanisms of stress tolerance in *R. soongorica*. Based on the putative functions of the identified genes, some important genes may be cloned. Moreover, the cloning of stress tolerance genes and determination of their expression patterns under drought stress may reveal attractive candidate genes and valuable information to improve drought stress tolerance of plants through genetic engineering.

## Materials and Methods

### Ethics Statement

*R. soongorica* is widely distributed in northwest China and is not listed as endangered or protected species. No specific permissions are required for sample collection in the Northern Mountain of Lanzhou, and that the field studies did not involve any endangered or protected species.

### Plant Materials and Drought Treatment

Seeds of *R. soongorica* were collected from the Northern Mountain of Lanzhou, Gansu, China (36°17′N, 103°48′E, 1700–1900 m elevation). Seeds were planted in the breeding base at the foothill. Three-year-old plants were transferred to a greenhouse under the following conditions: 150 µmol m^−2^s^−1^ (fluorescent tubes), photoperiod 16 h light/8 h darkness, day/night temperature of 25°C/20°C. The plants were washed with deionized water to remove surface contaminants. Soil attached to roots was also rinsed off with deionized water. The plants were then cultivated in 0.125×Murashige and Skoog medium solution (without vitamins and organics) with insufflating air for two weeks to accommodate the liquid environment during treatments. The samples were classified into untreated and PEG-treated groups. The untreated samples were maintained in cultivating conditions as previously described. For drought treatment, 15% PEG6000 was added to the culture solution of *R. soongorica*. All plants were treated for 12 h, from 6∶00 to 18∶00 local time. Leaves from each treatment were collected and immediately stored in liquid nitrogen for later use.

### DGE Library Preparation and Sequencing

Total RNA samples were isolated according to modified CTAB-method [34]. Quality and quantity analysis of total RNA, library construction, and sequencing were performed at the Beijing Genome Institute at Shenzhen, China. An extract of 8 µg of total RNA was obtained and treated with oligo (dT) magnetic bead adsorption to purify mRNA. Oligo (dT) was then used as primer in the synthesis of first- and second-strand cDNA. The 5′-ends of tags can be generated by two types of endonucleases, namely, NlaIII or DpnII. The bead-bound cDNA was subsequently digested with restriction enzyme NlaIII, which recognizes and severs CATG sites. Fragments other than 3′-cDNA fragments connected to oligo (dT) beads were washed away, and the Illumina adaptor 1 was ligated to the sticky 5′-end of the digested bead-bound cDNA fragments. The junction of the Illumina adaptor 1 and the CATG site constitutes the recognition site of MmeI, which is a type of endonuclease with separate recognition and digestion sites. MmeI breaks 17 bp downstream of the CATG site, producing tags with adaptor 1. After removing 3′ fragments with magnetic bead precipitation, the Illumina adaptor 2 was ligated to the 3′-ends of tags, thereby acquiring tags with different adaptors of both ends to form a tag library. After linear PCR amplification, fragments were purified by PAGE gel electrophoresis. During the quality control steps, Agilent 2100 Bioanalyzer and ABI Step One Plus Real-Time PCR System were used in the quantification and qualification of the sample library. Finally, the library was sequenced using Illumina HiSeq 2000 sequencer. The available raw data was then deposited in the NCBI Gene Expression Omnibus (GEO) database (http://www.ncbi.nlm.nih.gov/geo/query/acc.cgi?acc=GSE55412).

### Analysis and Mapping of DGE Tags

Sequencing-received raw image data were transformed by base calling into sequence data. Prior to mapping the readings from the reference database, all sequences were filtered to remove 3′ adaptor sequence, low-quality sequences (tags with unknown sequences ‘N’), tags with a copy number of one (probably sequencing error), and tags that were long or too short, leaving clean tags of 21 nt. For annotation, all clean tags were mapped in reference sequences, and a mismatch of only 1 bp was considered. We obtained the combined transcriptome of *R. soongorica* with (suffix is “_3″) and (suffix is “_A”) (http://www.ebi.ac.uk/arrayexpress/experiments/E-MTAB-1543/) [17], which was used as reference database. Clean tags were mapped in reference sequences. The clean reads mapped in reference database from multiple genes were filtered. The remaining clean tags were used as unambiguous clean tags. The number of unambiguous clean tags for each gene was calculated and normalized to TPM (transcripts per million clean tags).

### Identification and Functional Annotation of DEGs

We used a rigorous algorithm method to identify DEGs between two samples. False discovery rate (FDR) was applied to determine the threshold of P-value in multiple tests and analysis. The DEGs were obtained through FDR ≤0.001 and |log_2_Ratio| ≥1. Gene ontology (GO) and pathway classification was used to determine the possible functions of all DEGs by mapping the GO (http://www.geneontology.org/) and KEGG (http://www.genome.jp/kegg/) databases.

## Results

### Analysis of DGE Libraries

**Figure 1:**
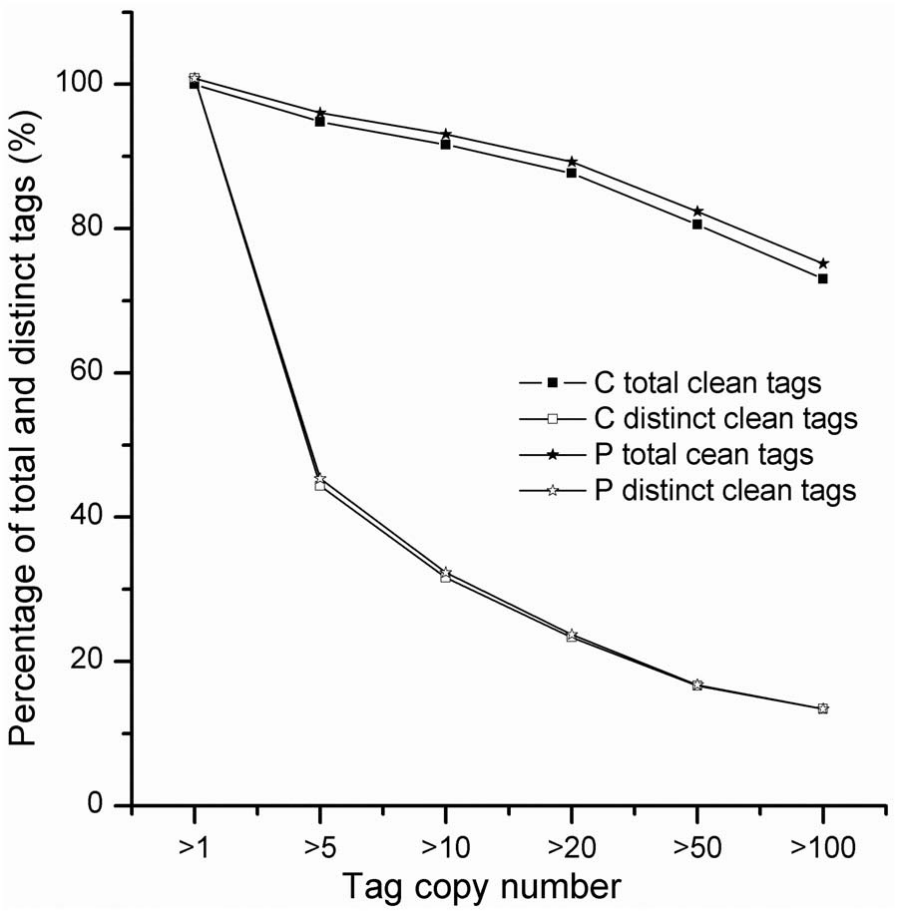
Distribution of total clean tag and distinct clean tag copy numbers from the libraries C and P. C represents the control group; P represents the PEG-treated group.

The Illumina platform was used to perform high throughput tag-seq analysis on *R. soongorica* to investigate transcriptome responses to PEG-induced drought stress. Total RNA isolated from untreated and PEG-treated groups were named as libraries C and P, respectively. The major characteristics of the two libraries are summarized in Table 1. An average of 7,231,417 total sequence tags per library was obtained with 466,957 distinct tag sequences from the two libraries. Prior to mapping tag sequences to the reference sequences, adaptor tags were filtered (low-quality tags and tags with one copy) and produced approximately 6,969,782 total clean sequence tags per library with 211,695 distinct clean tag sequences. The distributions of the total and distinct clean tag copy numbers showed highly similar tendencies in the two libraries (Figure 1). Among the distinct clean tags, more than 60% of the distinct clean tags had 2 to 5 copies, 34% of the distinct clean tags had 5 to 100 copies, and 4.5% had copy numbers higher than 100.

**Table 1:** Categorization and abundance of tags.

Summary		C	P
Raw tags	Total	7266032	7196802
	Distinct tags	445588	488325
Clean tags	Total number	7026443	6913120
	Distinct tag numbers	212338	211052
All tag mapping to gene	Total number	5869765	5356098
	Total % of clean tags	83.54%	77.48%
	Distinct tag numbers	110536	105096
	Distinct tag % of clean tags	52.06%	49.8%
Unambiguous tag mapping to gene	Total number	4261924	3722859
	Total % of clean tags	60.66%	53.85%
	Distinct tag numbers	85870	81776
	Distinct tag % of clean tags	40.44%	38.75%
All tag-mapped genes	Number	39354	38413
	% of ref genes	41.48%	40.49%
Unambigous tag-mapped genes	Number	27207	26381
	% of ref genes	28.68%	27.81%
Unknown tags	Total number	1156678	1557022
	Total % of clean tags	16.46%	22.52%
	Distinct tag numbers	101802	105956
	Distinct tag % of clean tags	47.94%	50.2%

Clean tags are tags that remained after filtering out dirty tags (low quality tags) from raw data. Distinct tags are different kinds of tags. Unambiguous tags are clean tags remaining after the removal of tags mapped to reference sequences from multiple genes. C represents the control group; P represents the PEG-treated group.

### Analysis of Tag Mapping

A reference gene database that included 94,878 sequences of the *R. soongorica* unigene was preprocessed for tag mapping. Among the sequences, genes with a CATG site accounted for 77.89%. To obtain the reference tags, all CATG+17 tags were used as gene reference tags. Finally, 218,791 total reference tag sequences with 182,184 unambiguous reference tags were obtained. Approximately 40.44% and 38.75% of the distinct clean tags were mapped unambiguously in the unigene database, and 47.94% and 50.2% of the distinct clean tags were not mapped to the unigene virtual tag database in libraries C and P, respectively (Table 1).

The sequencing saturation was analyzed in the two libraries to estimate whether the sequencing depth was sufficient for the transcriptome coverage. The genes that were mapped by all clean tags and unambiguous clean tags increased with the total number of tags. However, when the sequencing counts reached three million tags or higher, the number of detected genes was saturated (Figure S1). Given that Illumina sequencing can distinguish transcripts originating from both DNA strands and the strand-specific nature of the sequencing tags obtained, we found approximately 27% and 26% of distinct tags, which were mapped insense transcripts of libraries C and P, respectively (Figure S2). Approximately 25% and 24% of distinct tags perfectly matched antisense transcripts, which suggested that antisense genes play important roles in the transcriptional regulation of drought response in *R. soongorica*.

### Drought-caused Changes in the Global Gene Expression in *R. Soongorica*

To obtain the transcriptional changes in PEG-treated *R. soongorica*, a rigorous algorithm method was applied to identify DEGs from normalized DGE data using pairwise comparison between the two groups (C and P). Results showed that 1,325 genes had FDRs ≤0.001, and |log_2_Ratio| ≥1, which were declared to be the DEGs between the control and PEG-treated plants. Among these genes, 379 (28.6%) genes were up-regulated and 946 (71.4%) were down-regulated in response to drought stress. The expression of most genes was suppressed after drought treatment.

**Figure 2:**
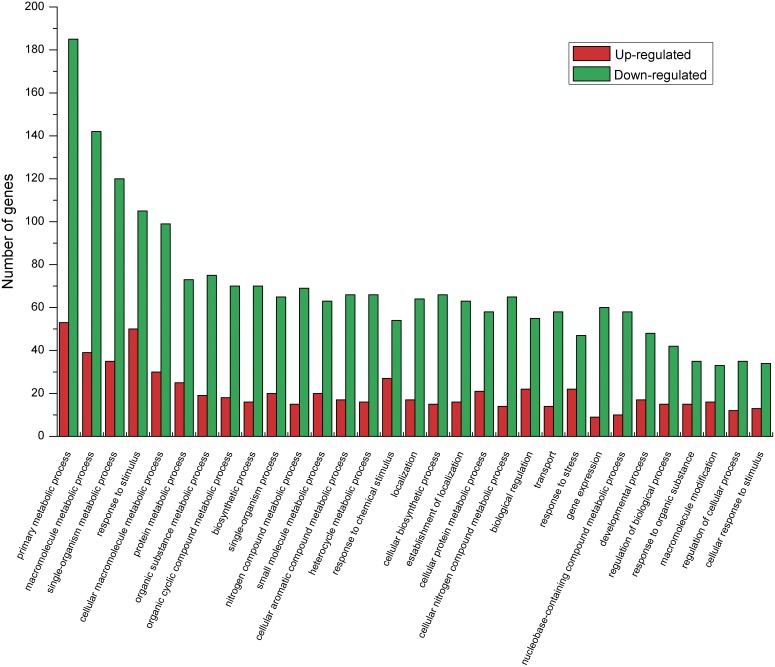
Gene classification based on gene ontology (GO) for DEGs in C and P libraries of *R. soongorica*. Only the biological processes were used for GO analysis. C represents the control group; P represents the PEG-treated group.

Functional annotation was performed to assign the genes of *R. soongorica* with GO terms. The main GO terms included “cellular component”, “molecular function”, and “biological process”. A total of 535 DEGs were annotated as “cellular component”. The categories of “cell part” and “cell” had the same proportion (88.0%), followed by “intracellular” and “intracellular part”, which accounted for 78.5% and 77.9% of assignments, respectively (Table S1). In addition, a total of 570 DEGs were annotated to “molecular function” terms. The categories “catalytic activity”, “organic cyclic compound binding”, and “heterocyclic compound binding” were prominent with 60.5%, 25.3% and 25.3% of assignments, respectively (Table S1). Furthermore, a total of 521 DEGs were annotated to “biological process” terms, in which 71.4% and 63.6% of the assignments belonged to “metabolic process” and “cellular process”, respectively, followed by “cellular metabolic process” and “primary metabolic process” (Figure 2).

To characterize the functional consequences associated with drought response, a pathway analysis of the DEGs based on the KEGG database was performed. The results indicated that genes participating in the biosynthesis of secondary metabolites, plant hormone signal transduction, plant-pathogen interaction, amino sugar and nucleotide sugar metabolism, and ubiquitin-mediated proteolysis were differentially expressed in PEG-treated group (Table S2).

### Genes Associated with the Major Functional Group were Affected by PEG-induced Drought Stress

The global gene expression results showed that a great number of genes were significantly affected by PEG-induced drought stress. We classified the DEGs into three groups.

Group A was composed of some regulatory proteins, such as transcription factors, protein kinase, and other signaling molecules (Table 2). Twenty genes encoding protein kinase, including CBL-interacting protein kinase (CIPK), threonine protein kinase, receptor-like protein kinase (RPK), mitogen-activated protein kinase (MAPK) cascade, phosphoenolpyruvate carboxylase kinase, and other kinases, were remarkably changed under drought stress. The results showed that six genes were up-regulated, and 14 genes were down-regulated. Meanwhile, a total of 14 drought-activated or repressed transcription factor genes were identified from several different families including WRKY, NAC, MYC, TCP, and bZIP. In our results, five transcription factors were identified as up-regulated, whereas nine transcription factors were repressed. The expression profiles exhibited that the majority of regulatory proteins were repressed after 12 h of drought treatment.

**Table 2:** Selected DEGs between the C and P libraries.

Funtional group	Unigene ID	Gene annotation	TPM-C	TPM-P	Log_2_Ratio (C/P)
**Protein kinases**					
	Unigene 9485_A	CBL-interacting protein kinase	2.99	12.87	2.11
	CL2201. Contig1_3	CBL-interacting protein kinase	25.9	59.45	1.20
	CL4525. Contig1_A	threonine protein kinase	4.98	15.62	1.65
	Unigene 2338_A	threonine protein kinase	2.85	7.96	1.48
	Unigene 14518_A	serine/threonine-protein kinase	78.13	35.73	-1.13
	Unigene 32052_3	receptor-like protein kinase	2.56	0.01	-8
	Unigene 39297_A	receptor-like protein kinase	2.13	0.01	-7.73
	Unigene 52174_A	receptor-like protein kinase	6.26	1.3	-2.27
	CL2777. Contig2_3	receptor-like protein kinase	36.43	14.9	-1.29
	CL6483. Contig1_3	receptor-like protein kinase	16.94	7.67	-1.14
	Unigene 43240_A	receptor-like protein kinase	76.71	38.19	-1.01
	Unigene 21624_3	mitogen-activated protein kinase kinase kinase	7.26	1.59	-2.19
	CL1583.Contig2_A	mitogen-activated protein kinase kinase kinase	7.26	1.59	-2.19
	Unigene 9581_3	mitogen-activated protein kinase 2	25.62	10.7	-1.26
	Unigene 633_3	AMP-activated protein kinase	24.76	10.7	-1.21
	Unigene 21618_3	calcineurin B-like protein-interacting protein kinase	25.33	12.01	-1.08
	Unigene 10286_A	phosphoenolpyruvate carboxylase kinase	0.57	4.63	3.02
	Unigene 27156_3	pyruvate, phosphate dikinase	35.44	85.2	1.27
	CL4464. Contig2_3	pyruvate kinase	85.82	29.36	-1.55
	Unigene 50230_A	histidine kinase	40.28	17.94	-1.67
**Transcription factors**					
	Unigene 30342_3	WRKY transcription factor 7	4.7	14.75	1.65
	Unigene 10063_3	WRKY transcription factor 6–1	1.99	0.01	-7.64
	Unigene 27699_3	MYC protein	14.23	47.74	1.75
	CL7944. Contig1_3	NAC domain-containing protein	14.23	4.48	-1.67
	Unigene 31521_3	NAC domain-containing protein	12.52	4.19	-1.58
	Unigene 25674_3	transcription factor TCP20-like	13.66	33.7	1.30
	CL7341. Contig1_3	transcription factor TCP20-like	45.26	16.92	-1.42
	Unigene 22142_3	transcription factor TCP7-like	31.88	15.04	-1.08
	Unigene 21994_3	DNA binding protein	6.55	14.32	1.13
	Unigene 23673_3	transcription factor bZIP124	51.24	20.83	-1.29
	Unigene 1336_3	transcription factor bZIP12	4.7	11.57	1.29
	Unigene 28584_3	transcription factor UNE12	37.86	12.01	-1.66
	CL8097. Contig1_A	transcription factor UNE12	8.11	3.18	-1.35
	CL5168. Contig1_3	transcription factor UNE12	12.95	5.35	-1.28
**Flavonoids biosynthesis**					
	CL93. Contig1_A	UDP-glucose: glucosyltransferase	22.06	50.19	1.19
	CL1676. Contig1_A	Flavonol sulfotransferase-like protein	3.56	9.4	1.40
	CL2370. Contig2_3	flavonoid 3′-hydroxylase	10.39	25.6	1.30
	Unigene 1473_3	flavonoid 3′-hydroxylase	84.96	34.72	-1.29
	Unigene 19573_A	dihydroflavonol 4-reductase	1.14	17.65	3.95
**LEA proteins**					
	CL4558. Contig1_3	late embryogenesis abundant protein	5.84	12.73	1.12
	Unigene 442_3	late embryogenesis abundant protein group 9	7.54	22.57	1.58
**Heat shock proteins**					
	Unigene 18824_A	mitochondrial small heat shock protein	3.84	10.99	1.51
	CL12936. Contig1_A	heat shock protein 17.5 cytosolic class II	1.85	8.25	2.15
**aquaporin**					
	Unigene 39258_A	aquaporin	1.71	9.69	2.50
	Unigene 19242_A	aquaporin	426.96	176.77	-1.27
**Proline transporter**					
	CL1109. Contig2_A	proline transporter	7.54	27.34	1.86
**Cold shock protein**					
	Unigene 233_3	cold shock protein	11.53	58.44	2.34
**low temperature-induced protein**					
	Unigene 24454_A	low temperature-induced protein	0.01	2.03	7.67
**Dehydration-induced proteins**					
	Unigene 922_A	dehydration-induced proteins	10.67	21.99	1.04
**universal stress protein**					
	Unigene 25385_3	universal stress protein	93.22	263.56	1.50
**Resistance protein**					
	CL8753. Contig4_3	disease resistance protein	1.85	7.23	1.97
	CL6875. Contig2_3	disease resistance protein	0.01	2.03	7.67
	CL5118. Contig1_3	disease resistance protein	4.7	1.01	-2.22
	Unigene 26781_3	natural resistance-associated macrophage protein	1.28	16.49	3.69
	Unigene 28389_3	nematode resistance protein	36.86	77.68	1.08
**defensin precursor**					
	Unigene 26068_3	defensin precursor	20.92	87.08	2.06
**chitinase**					
	CL630. Contig2_3	class IV chitinase	3.7	67.84	4.20
	CL5695. Contig1_A	chitinase	6.4	41.8	2.71
	Unigene 30551_3	class IV chitinase	13.52	6.08	-1.15
	CL630. Contig3_3	class IV chitinase	60.91	27.63	-1.14

C represents the control group; P represents the PEG-treated group. The DEGs was selected through FDR ≤0.001 and |log_2_Ratio| ≥1.

Group B was composed of 13 functional protein genes, which included genes involved in flavonoid biosynthesis (6 DEGs), LEA protein (2 DEGs), small heat shock protein (sHSP) (2 DEGs), aquaporin (2 DEGs), and proline transporters (1 DEG) (Table 2). Contrary to the expression of regulatory protein genes, most of the functional protein genes were up-regulated after 12 h of drought treatment.

Group C was composed of 14 proteins that were annotated as low-temperature-induced protein (1 DEG), dehydration-induced protein (1 DEG), defensing precursor (1 DEG), resistance protein (4 DEG), universal stress protein (1 DEG), and proteins involved in chitinase biosynthesis (4 DEGs) (Table 2). Similar to the alteration of functional protein genes, the expression of these 14 genes also exhibited a general upward trend.

## Discussion

Plants, which are sessile organisms, try to respond and adapt to stress by altering their gene expression patterns when they are subjected to adverse environments [7]. Therefore, the genes that showed different expressions are probably involved in stress response. Shinozaki and Yamaguchi-Shinozaki [10] reported that the drought-inducible genes identified can be classified into regulatory and functional proteins. The regulatory proteins include various transcription factors, protein phosphatases, protein kinases, and other signaling molecules that participate in further regulation of signal transduction and downstream gene expression. The functional proteins are composed of molecules, such as chaperones, water channel proteins, LEA proteins, mRNA-binding proteins, sugar and proline transporters, detoxification enzymes, and various proteases. In the present study, the DEGs between the leaves of drought-stressed and unstressed *R. soongorica* were investigated to conduct a global analysis of the transcriptomic response, which can facilitate our understanding of drought tolerance mechanisms. In our results, 1325 DEGs with 379 up-regulated and 946 down-regulated genes were detected. Our results are similar to that of Fan et al. [6], who also reported that down-regulated genes were more abundant than up-regulated in soybean leaves after drought exposure. Functional annotation analysis results indicated that many drought-inducible genes with various functions were identified in *R. soongorica*, including a number of regulatory proteins, functional proteins, and other inducible proteins in response to drought stress.

Regulatory proteins are involved in signal transduction pathways and in controlling the expression of functional genes in stress responses. In higher plants, many regulatory genes, such as those encoding protein kinases and transcription factors, are induced by environmental signals or stresses [35]. In this study, 20 DEGs encoding protein kinases were detected after drought treatment. Various protein kinases including CIPK protein, threonine protein kinase, MAPK, calcium-dependent protein kinases, and RPK are important regulatory proteins in the regulation of some stress-inducible genes [36]. Several experiments have demonstrated that protein kinase signaling conferred plant resistance to stress by regulating downstream functional genes [37], [38]. In addition, protein kinase also functions in regulating transcription factors [39]. A total of 14 genes encoding transcription factors, including WRKY, NAC, MYC, TCP, and basic region leucine zipper (bZIP), were also significantly differentially expressed. Transcription factors are important in regulating plant responses to biotic and abiotic stresses [40]. Altering the expression of certain transcription factors can greatly change the expression of a number of stress-related target genes that influence plant stress tolerance in transgenic plants [9], [10]. Thus, the alteration of protein kinases and transcription factors may function in the signal transduction process by regulating the expression of various functional genes in response to drought stress.

Aside from signaling transduction, plants adapt to adverse environment through the activation or up-regulation of genes that directly function in stress resistance [7]. Flavonoids are a class of secondary metabolites with diverse functions in growth, development, reproduction, and are also involved in diverse stress responses in plants. Flavonoids accumulate rapidly and play a protective role when plants are exposed to abiotic stress [41]. In recent years, several genes encoding important enzymes, such as phenylalanine ammonia-lyase, F3H, dihydroflavonol 4-reductase (DFR), and anthocyanin synthase, which are involved in the flavonoid biosynthetic pathway, were found to be up-regulated in response to different kinds of stresses, such as salinity, UV-B irradiation, water deficit, and nitrogen stress [41]–[44]. Increased expression of genes involved in the flavonoid pathway was detected in our previous study on *R. soongorica* [16]. In the present study, most DEGs encoding key enzymes involved in flavonoid biosynthesis were also up-regulated, especially DEGs that encode UFGT, DFR, and F3′H. These findings are consistent with the results of other studies conducted on birch, potato, and rice after stress exposure [45]–[47]. The up-regulated genes may result in accumulation of flavonoids. Flavonoids may serve as signaling molecules in signaling cascades. Flavonoids also affect cellular function and modulate gene expression in response to adverse conditions [48]. Agati et al. [49] found that flavonoids directly scavenged the ROS brought about by stress. Therefore, up-regulation of flavonoid pathway genes may enhance the protective role of *R. soongorica*, thus leading to better drought tolerance.

Secondary metabolites and stress-responsive proteins, such as LEA proteins, accumulate naturally in the seeds of some desiccation-tolerant plants, and are also induced in vegetative tissues when subjected to water-limited conditions [50], [51]. LEA proteins, largely attributed to their extreme hydrophilic nature, have also been implicated in detoxification, sequestration of ions, maintenance of protein or membrane structure, binding water, and operation as molecular chaperones during dehydration [52]. In this study, two DEGs were identified as up-regulated LEA proteins. Our results are in agreement with the findings of Gechev et al. [53], who found high expression of LEA genes during drought and desiccation in leaves of *Haberlea rhodopensis*. We concluded that dehydration-induced expression of LEA transcripts is a common response in resurrection and desiccation sensitive plants [54]. Improved resistance to drought and salinity was also observed in transgenic rice when LEA was overexpressed [55]. Expression of LEA proteins in yeast confers improved resistance to high salinity and freezing [56]. These studies demonstrated that LEA proteins functions in binding water, maintenance of protein or membrane structure, or operation as molecular chaperones to enhance tolerance to dehydration, whereas few additional clues to the mechanism of protein function are provided.

Plant HSPs facilitate protein folding or assembly under diverse developmental and adverse environmental conditions. In this study, two small HSPs (sHSPs) encoding DEGs showed up-regulation under drought stress. One sHSP was annotated to mitochondrial sHSP (MT-sHSP), and the other was annotated as cytosolic sHSP17.5. Although the precise functional mechanism of sHSPs is still unclear, in vivo studies have shown that sHSPs function as molecular chaperones in stressful conditions and in normal development [57]. Overproduction of sHSP increased drought tolerance in transgenic rice seedlings [58]. MT-sHSP protects the electron transport complex I and prevents damage by ROS [59]. Lee et al. [60] found that transgenic plants with over-expression of MT-sHSP efficiently maintained membrane stability by scavenging ROS, and were conferred with enhanced tolerance to salinity. Ectopic expression of cytosolic sHSP 17.1 in *Arabidopsis thaliana* exhibited higher resistance to drought [61]. In a study conducted by Sato et al. [62], sHSP 17.5 showed molecular chaperone activity in vitro and represented an example of an HSP capable of protecting cells against thermal extremes. Therefore, these results indicated that the sHSPs may play roles in scavenging ROS or functioning as molecular chaperone to respond to drought stress.

The DEGs encoding aquaporins and proline transporter were also identified. Aquaporins are channel proteins of intracellular and plasma membranes that function in transporting water, small solutes (urea, boric acid, and silicic acid), and gases (ammonia and carbon dioxide). A specific role for aquaporins in embolism refilling and recovery of stem axial conductance after drought was proposed in grapevine [63]. In our results, two aquaporin genes were identified. One aquaporin gene was up-regulated, whereas the other gene was down-regulated. An overall tendency of down regulation for aquaporin gene was observed. However, up regulation of certain aquaporin transcripts was also detected in rice and Arabidopsis leaves [64], [65]. Therefore, transcriptional control of aquaporins in drought-stressed leaves appears to be more complex [66]. Interestingly, transport of proline may also be affected by water deficit. A gene encoding proline transporter was up-regulated under drought stress in the present study, which is in agreement with the result of Rentsch et al. [67], who also observed the induction of a specific proline transporter in Arabidopsis under water deficit. The DEGs encoding aquaporins and proline transporter in *R. soongorica* may serve as an adaptive strategy by regulating water and proline distribution throughout the whole plant.

Moreover, in addition to two dehydration-induced 19 homologs, proteins that can be induced by other stresses were also detected in the study and annotated to low-temperature-induced (LT) protein, cold shock protein (CSP), defensin precursor, chitinase, disease resistance protein, natural resistance-associated macrophage protein, nematode resistance protein, and universal stress protein. LT proteins and CSPs are commonly induced by cold stress. Various genes are induced by both drought and cold stress, suggesting the existence of crosstalk between drought and cold-stress signaling pathways [10]. Defensins, chitinase, disease resistance proteins, natural resistance-associated macrophage protein, and nematode resistance protein were all found to be involved in the defense against phytopathogens [68]–[72]. Therefore, the identification of the genes in this study suggested that a crosstalk between drought and pathogen attack. The universal stress proteins can confer the ability to respond and adapt to environmental changes [73]. The identification of all these genes suggests crosstalk between responsive genes or pathways and multiple abiotic or even biotic stresses [23]. Moreover, these identified genes may provide gene resources for understanding the drought tolerance mechanism of *R. soongorica*.

Among the identified DEGs, most regulatory proteins were down-regulated, whereas the majority of functional proteins were up-regulated. The different expression patterns shown by the two groups are interesting and worthy to be explored. Therefore, we tried to discuss the probable interaction of regulatory proteins and functional proteins. Most gene regulators exhibit either antecedent or simultaneous change in the expression level when compared with their targets [74]. One reason is that it takes time for the regulator gene to express its protein product and affect (directly or indirectly) the transcription of its target gene [75]. Other reasons may have been caused by the different mRNA half-lives of the regulator and target genes, and the different profiles between regulator genes and functional genes may also indicate a feedback loop, where a target gene can regulate its regulator [76]. Given that signaling pathways are complex dynamic events that occur over time, single time point expression profiles in our study are insufficient to elucidate temporal events. The different expression profiles of regulatory and functional proteins observed in this study are interesting. Expression profiling experiment with a series of time points should be performed to elucidate this problem in further studies.

## Conclusions

Based on the putative function analysis of annotated DEGs, DEGs are classified into three groups. The first group, which includes protein kinases (CIPK and threonine protein kinase) and transcription factors (WRKY, MYC and TCP), regulates signal transduction. The second group includes genes involved in the biosynthesis of flavonoids in scavenging ROS, sHSP, LEA proteins, aquaporins, and proline transporter that represents functional genes that directly enhance drought-stress tolerance. The sHSP maintains protein or membrane structure and act as molecular chaperones that protect cells from injury under drought stress. Aquaporins and proline transporter regulates water or proline distribution throughout the whole plant. The third group contains proteins induced by other stress factors, which suggests that a crosstalk between different stresses. In conclusion, the tolerant ability of *R. soongorica* may be gained through effective signal transduction and enhanced protection of functional proteins to reestablish cellular homeostasis.

## Supporting Information

Figure S1 Sequencing saturation analysis of the two libraries. C represents the control group; P represents the PEG-treated group.
(TIF)

Figure S2 Mapping of distinct clean tags in the two DGE libraries. C represents the control group; P represents the PEG-treated group.
(TIF)

Table S1 Gene classification based on gene ontology (GO) for DEGs in C and P libraries of *R. soongorica*. C represents the control group; P represents the PEG-treated group.
(XLSX)

Table S2 KEGG analysis for DEGs in C and P libraries of *R. soongorica*. C represents the control group; P represents the PEG-treated group.
(XLSX)

## References

[1] Okcu G, Kaya MD, Atak M (2005) Effects of salt and drought stresses on germination and seedling growth of pea (*Pisumsativum* L.). Turk J Agric For 29: 237–242.

[2] Lawson T, Oxborough K, Morison JIL, Baker NR (2003) The responses of guard and mesophyll cell photosynthesis to CO_2_, O_2_, light, and water stress in a range of species are similar. J Exp Bot 54: 1743–52. 12773521 10.1093/jxb/erg186

[3] Dat JF (1998) Parallel changes in H_2_O_2_ and catalase during thermotolerance induced by salicylic acid or heat acclimation in mustard seedlings. Plant Physiol 116: 1351–1357. 9536052 10.1104/pp.116.4.1351 PMC35042

[4] Liu YB, Wang G, Liu JY, Zhao X, Tan HJ, et al (2007) Anatomical, morphological and metabolic acclimation in the resurrection plant *Reaumuria soongorica* during dehydration and rehydration. J Arid Environ 70: 183–194.

[5] McDowell N, Pockman WT, Allen CD, Breshears DD, Cobb N, et al (2008) Mechanisms of plant survival and mortality during drought: why do some plants survive while others succumb to drought? New Phytol 178: 719–739. 18422905 10.1111/j.1469-8137.2008.02436.x

[6] Fan XD, Wang JQ, Yang N, Dong YY, Liu L, et al (2013) Gene expression profiling of soybean leaves and roots under salt, saline–alkali and drought stress by high-throughput Illumina sequencing. Gene 512: 392–402. 23063936 10.1016/j.gene.2012.09.100

[7] Hazen SP, Pathan MS, Sanchez A, Baxter I, Dunn M, et al (2005) Expression profiling of rice segregating for drought tolerance QTLs using a rice genome array. Funct Integr Genomic 5: 104–116. 10.1007/s10142-004-0126-x 15480887

[8] Seki M, Kameiy A, Yamaguchi-Shinozakiz K, Shinozaki K (2003) Molecular responses to drought, salinity and frost: common and different paths for plant protection. Curr Opin Biotech 14: 194–199. 12732320 10.1016/s0958-1669(03)00030-2

[9] Yamaguchi-Shinozaki K, Shinozaki K (2005) Organization of *cis*-acting regulatory elements in osmotic- and cold-stress-responsive promoters. Trends Plant Sci 10: 88–94. 15708346 10.1016/j.tplants.2004.12.012

[10] Shinozaki K, Yamaguchi-Shinozaki K, Seki M (2003) Regulatory network of gene expression in the drought and cold stress responses. Curr Opin Plant Biol 6: 410–417. 12972040 10.1016/s1369-5266(03)00092-x

[11] Ma JY, Chen T, Qiang WY, Wang G (2005) Correlations between foliar stable carbon isotope composition and environmental factors in desert plant *Reaumuria soongorica* (Pall.) Maxim. J Intergr Plant Biol 47: 1065–1073.

[12] Liu YB, Zhang TG, Wang J (2008) Photosynthesis and metabolite leaves levels in dehydrating leaves of *Reaumuria soongorica* . Acta Biol Cracov Bot 50: 19–26.

[13] Bai J, Gong CM, Chen K, Kang HM, Wang G (2009) Examination of antioxidative systems responses in the different phases of drought stress and during recovery in desert plant *Reaumuria soongorica* (Pall.) Maxim. J Plant Biol 52: 417–425.

[14] Wang XH, Zhang T, Wen ZN, Xiao HL, Yang ZJ, et al (2011) The chromosome number, karyotype and genome size of the desert plant diploid *Reaumuria soongorica* (Pall.) Maxim. Plant Cell Rep 30: 955–964. 21327391 10.1007/s00299-011-1020-3

[15] Liu YB, Li XR, Tan HJ, Liu ML, Zhao X, et al (2010) Molecular characterization of RsMPK2, a C1 subgroup mitogen-activated protein kinase in the desert plant *Reaumuria soongorica* . Plant Physiol Biochem 48: 836–844. 20833058 10.1016/j.plaphy.2010.07.001

[16] Liu ML, Li XR, Liu YB, Cao B (2013) Regulation of flavanone 3-hydroxylase gene involved in the flavonoid biosynthesis pathway in response to UV-B radiation and drought stress in the desert plant, *Reaumuria soongorica* . Plant Physiol Biochem 73: 161–167. 24121417 10.1016/j.plaphy.2013.09.016

[17] Shi Y, Yan X, Zhao PS, Yin HX, Zhao X, et al (2013) Transcriptomic analysis of a tertiary relict plant, extreme xerophyte *Reaumuria soongorica* to identify genes related to drought adaptation. PLoS One 8: e63993. 23717523 10.1371/journal.pone.0063993 PMC3662755

[18] Diatchenko L, Laue YF, Campbell AP, Chenchik A, Moqadam F, et al (1996) Suppression subtractive hybridization: a method for generating differentially regulated or tissue-specific cDNA probes and libraries. P Natl Acad Sci USA 93: 6025–6030. 10.1073/pnas.93.12.6025 PMC39182 8650213

[19] Lertkiatmongkol P, Pethuan S, Jirakanjanakit N, Rongnoparut P (2010) Transcription analysis of differentially expressed genes in insecticide-resistant *Aedes aegypti* mosquitoes after deltamethrin exposure. J Vector Ecol 35: 197–203. 20618667 10.1111/j.1948-7134.2010.00047.x

[20] Asakura T, Tamura T, Terauchi K, Narikawa T, Yagasaki K, et al (2012) Global gene expression profiles in developing soybean seeds. Plant Physiol Biochem 52: 147–153. 22245912 10.1016/j.plaphy.2011.12.007

[21] Caruso A, Chefdor F, Carpin S, Depierreux C, Delmotte FM, et al (2008) Physiological characterization and identification of genes differentially expressed in response to drought induced by PEG 6000 in *Populus Canadensis* leaves. J Plant Physiol 165: 932–941. 17928100 10.1016/j.jplph.2007.04.006

[22] Chen FY, Liu HP, Bo J, Ren HL, Wang KJ (2010) Identification of genes differentially expressed in hemocytes of *Scylla* paramamosain in response to lipopolysaccharide. Fish Shellfish Immun 28: 167–177. 10.1016/j.fsi.2009.10.017 19854276

[23] Zhou XG, Wu FZ (2009) Differentially expressed transcripts from cucumber (*Cucumis sativus* L.) root upon inoculation with *Fusarium oxysporum* f. sp. *cucumerinum* Owen. Physiol Mol Plant P 74: 142–150.

[24] Da XL, Yu KQ, Shen SH, Zhang YJ, Wu JX, et al (2012) Identification of differentially expressed genes in a spontaneous altered leaf shape mutant of the navel orange [*Citrus sinensis* (L.) Osbeck]. Plant Physiol Biochem 56: 97–103. 22609459 10.1016/j.plaphy.2012.04.008

[25] Hong LZ, Li J, Schmidt-Küntzel A, Warren WC, Barsh GS (2011) Digital gene expression for non-model organisms. Genome Res 21: 1905–1915. 21844123 10.1101/gr.122135.111 PMC3205575

[26] Gao P, Chen AL, Zhao QL, Shen XJ, Qiu ZY, et al (2013) Differentially expressed genes in the ovary of the sixth day of pupal “Ming” lethal egg mutant of silkworm, *Bombyxmori* . Gene 527: 161–166. 23769927 10.1016/j.gene.2013.05.049

[27] Cheng LB, Li SY, Xu XY, Hussain J, Yin JJ, et al (2013) Identification of differentially expressed genes relevant to corm formation in *Sagittaria triflia* . PLoS One 8: e54573. 23359383 10.1371/journal.pone.0054573 PMC3554737

[28] Qin YF, Fang HM, Tian QN, Bao ZX, Lu P, et al (2011) Transcriptome profiling and digital gene expression by deep-sequencing in normal/regenerative tissues of planarian *Dugesia japonica* . Genomics 97: 364–371. 21333733 10.1016/j.ygeno.2011.02.002

[29] Chen S, Jiang J, Li HY, Liu GF (2012) The salt-responsive transcriptome of *Populussimonii × Populusnigra* via DGE. Gene 504: 203–212. 22634611 10.1016/j.gene.2012.05.023

[30] Wei MM, Song MZ, Fan SL, Yu SX (2013) Transcriptomic analysis of differentially expressed genes during anther development in genetic male sterile and wild type cotton by digital gene-expression profiling. BMC genomics 14: 97. 23402279 10.1186/1471-2164-14-97 PMC3599889

[31] Albouyeh R, Farzaneh N, Bohlmann J, Ritland K (2010) Multivariate analysis of digital gene expression profiles identifies a xylem signature of the vascular tissue of white spruce (*Piceaglauca*). Tree Genet Genomes 6: 601–611.

[32] Jiang JJ, Shao YL, Du K, Ran LP, Fang XP, et al (2013) Use of digital gene expression to discriminate gene expression differences in early generations of resynthesized *Brassica napus* and its diploid progenitors. BMC Genomics 14: 72. 23369045 10.1186/1471-2164-14-72 PMC3608150

[33] Nishiyama T, Miyawaki K, Ohshima M, Thompson K, Nagashima A, et al (2012) Digital gene expression profiling by 5-end Sequencing of cDNAs during reprogramming in the moss physcomitrella patens. PLoS One 7: e36471. 22574165 10.1371/journal.pone.0036471 PMC3344888

[34] Wang XH, Xiao HL, Chen GX, Zhao X, Huang CH, et al (2011) Isolation of high-quality RNA from *Reaumuria soongorica*, a desert plant rich in secondary metabolites. Mol Biotechnol 48: 165–172. 21136208 10.1007/s12033-010-9357-3

[35] Shinozaki K, Yamaguchi-Shinozaki K (1996) Molecular responses to drought and cold stress. Curr Opin Biotech 7: 161–167. 8791336 10.1016/s0958-1669(96)80007-3

[36] Zhu JK (2002) Salt and drought stress signal transduction in plants. Annu Rev Plant Biol 53: 247–273. 12221975 10.1146/annurev.arplant.53.091401.143329 PMC3128348

[37] Boller T, Felix G (2009) A renaissance of elicitors: perception of microbe-associated molecular patterns and danger signals by pattern-recognition receptors. Annu Rev Plant Biol 60: 379–406. 19400727 10.1146/annurev.arplant.57.032905.105346

[38] Lacombe S, Rougon-Cardoso A, Sherwood E, Peeters N, Dahlbeck D, et al (2010) Interfamily transfer of a plant pattern-recognition receptor confers broad-spectrum bacterial resistance. Nat Biotechnol 28: 365–369. 20231819 10.1038/nbt.1613

[39] Tena G, Boudsocq M, Sheen J (2011) Protein kinase signaling networks in plant innate immunity. Curr Opin Plant Biol 14: 519–529. 21704551 10.1016/j.pbi.2011.05.006 PMC3191242

[40] Chen WQ, Provart NJ, Glazebrook J, Katagiri F, Chang HS, et al (2002) Expression profile matrix of Arabidopsis transcription factor genes suggests their putative functions in response to environmental stresses. Plant Cell 14: 559–574. 11910004 10.1105/tpc.010410 PMC150579

[41] Castellarin SD, Matthews MA, Gaspero GD, Gambetta GA (2007) Water deficits accelerate ripening and induce changes in gene expression regulating flavonoid biosynthesis in grape berries. Planta 227: 101–112. 17694320 10.1007/s00425-007-0598-8

[42] Park JS, Choung MG, Kim JB, Hahn BS, Kim JB, et al (2007) Genes up-regulated during red coloration in UV-B irradiated lettuce leaves. Plant Cell Rep 26: 507–516. 17086420 10.1007/s00299-006-0255-x

[43] Walia H, Wilson C, Condamine P, Liu X, Ismail AM, et al (2005) Comparative transcriptional profiling of two contrasting rice genotypes under salinity stress during the vegetative growth stage. Plant Physiol 139: 822–835. 16183841 10.1104/pp.105.065961 PMC1255998

[44] Han YP, Vimolmangkang S, Soria-Guerra RE, Rosales-Mendoza S, Zheng D, et al (2010) Ectopic expression of apple F3′H genes contributes to anthocyanin accumulation in the Arabidopsis tt7 mutant grown under nitrogen stress. Plant Physiol 153: 806–820. 20357139 10.1104/pp.109.152801 PMC2879788

[45] Morales LO, Tegelberg R, Brosché M, Keinänen M, Lindfors A, et al (2010) Effects of solar UV-A and UV-B radiation on gene expression and phenolic accumulation in *Betulapendula* leaves. Tree Physiol 30: 923–934. 20519675 10.1093/treephys/tpq051

[46] André CM, Schafleitner R, Legay S, Lefèvre I, Aliaga CAA, et al (2009) Gene expression changes related to the production of phenolic compounds in potato tubers grown under drought stress. Phytochemistry 70: 1107–1116. 19664789 10.1016/j.phytochem.2009.07.008

[47] Lenka SK, Katiyar A, Chinnusamy V, Bansal KC (2011) Comparative analysis of drought-responsive transcriptome in Indica rice genotypes with contrasting drought tolerance. Plant Biotechnol J 9: 315–327. 20809928 10.1111/j.1467-7652.2010.00560.x

[48] Williams RJ, Spencer JPE, Rice-Evans C (2004) Flavonoids: antioxidants or signaling molecules? Free Radical Biol Med 26: 838–849. 10.1016/j.freeradbiomed.2004.01.001 15019969

[49] Agati G, Biricolti S, Guidi L, Ferrni F, Fini A, et al (2011) The biosynthesis of flavonoids is enhanced similarly by UV radiation and root zone salinity in L. *vulgare* leaves. J Plant Physiol 168: 204–212. 20850892 10.1016/j.jplph.2010.07.016

[50] Battaglia M, Olvera-Carrillo Y, Garciarrubio A, Campos F, Covarrubias AA (2008) The enigmatic LEA proteins and other hydrophilins. Plant Physiol 148: 6–24. 18772351 10.1104/pp.108.120725 PMC2528095

[51] Tunnacliffe A, Wise MJ (2007) The continuing conundrum of LEA proteins. Naturwissenschaften 94: 791–812. 17479232 10.1007/s00114-007-0254-y

[52] Bray EA (1996) Plant responses to water deficit. Trends Plant Sci 96: 1360–1385.

[53] Gechev TS, Benina M, Obata T, Tohge T, Sujeeth N, et al (2013) Molecular mechanisms of desiccation tolerance in the resurrection glacial relic *Haberlea rhodopensis* . Cell Mol Life Sci 70: 689–709. 22996258 10.1007/s00018-012-1155-6 PMC11113823

[54] Challabathula D, Bartels D (2013) Desiccation tolerance in resurrection plants: new insights from transcriptome, proteome and metabolome analysis. Front Plant Sci 4: 482. 24348488 10.3389/fpls.2013.00482 PMC3842845

[55] Xu D, Duan X, Wang B, Hong B, Ho THD, et al (1996) Expression of a late embryogenesis abundant protein gene, HVA1, from barley confers tolerance to water deficit and salt stress in transgenic rice. Plant Physiol 110: 249–257. 12226181 10.1104/pp.110.1.249 PMC157716

[56] Imai R, Chang L, Ohta A, Bray EA, Takagi M (1996) A lea-class gene of tomato confers salt and freezing tolerance when overexpressed in *Saccharomyces* cerevisiae. Gene 170: 243–248. 8666253 10.1016/0378-1119(95)00868-3

[57] Sun WN, Montagu MV, Verbruggen N (2002) Small heat shock proteins and stress tolerance in plants. Biochim Biophys Acta 1577: 1–7. 12151089 10.1016/s0167-4781(02)00417-7

[58] Sato Y, Yokoya S (2008) Enhanced tolerance to drought stress in transgenic rice plants overexpressing a small heat-shock protein, sHSP17.7. Plant Cell Rep 27: 329–334. 17968552 10.1007/s00299-007-0470-0

[59] Banzet N, Richaud C, Deveaux Y, Kazmaier M, Gagnon J, et al (1998) Accumulation of small heat shock proteins, including mitochondrial HSP22, induced by oxidative stress and adaptive response in tomato cells. Plant J 13: 519–527. 9680997 10.1046/j.1365-313x.1998.00056.x

[60] Lee KW, Cha JY, Kim KH, Kim YG, Lee BH, et al (2012) Overexpression of alfalfa mitochondrial HSP23 in prokaryotic and eukaryotic model systems confers enhanced tolerance to salinity and arsenic stress. Biotechnol Lett 34: 167–174. 22127759 10.1007/s10529-011-0750-1 PMC3235403

[61] Zhang L, Gao YK, Pan HT, Hu WJ, Zhang QX (2013) cloning and characterization of a *Primula* heat shock protein gene, PfHSP17.1, which confers heat, salt and drought tolerance in transgenic *Arabidopsis thaliana* . Acta Physiol Plant 35: 3191–3200.

[62] Sato A, Allona I, Collada C, Guevara MA, Casado R, et al (1999) Heterologous expression of a plant small heat-shock protein enhances Escherichia coli viability under heat and cold stress. Plant Physiol 120: 521–528. 10364403 10.1104/pp.120.2.521 PMC59290

[63] Lovisolo C, Schubert A (2006) Mercury hinders recovery of shoot hydraulic conductivity during grapevine rehydration: evidence from a whole-plant approach. New Phytol 172: 469–478. 17083677 10.1111/j.1469-8137.2006.01852.x

[64] Alexandersson E, Fraysse L, Sjövall-Larsen S, Gustavsson S, Fellert M, et al (2005) Whole gene family expression and drought stress regulation of aquaporins. Plant Mol Biol 59: 469–484. 16235111 10.1007/s11103-005-0352-1

[65] Guo L, Wang ZY, Lin H, Cui WE, Chen J, et al (2006) Expression and functional analysis of the rice plasma-membrane intrinsic protein gene family. Cell Res 16: 277–286. 16541126 10.1038/sj.cr.7310035

[66] Maurel C, Verdoucq L, Luu DT, Santoni V (2008) Plant aquaporins: membrane channels with multiple integrated functions. Annu Rev Plant Biol 59: 595–624. 18444909 10.1146/annurev.arplant.59.032607.092734

[67] Rentsch D, Hirner B, Schmelzer E, Frommer WB (1996) Salt stress-induced proline transporters and salt stress-repressed broad specificity amino acid permeases identified by suppression of a yeast amino acid permease-targeting mutant. Plant Cell 8: 1437–1446. 8776904 10.1105/tpc.8.8.1437 PMC161269

[68] Thomma BP, Cammue BP, Thevissen K (2002) Plant defensins. Planta 216: 193–202. 12447532 10.1007/s00425-002-0902-6

[69] Hamid R, Khan MA, Ahmad M, Ahmad MM, Abdin MZ, et al (2013) Chitinases: an update. J Pharm Bioallied Sci 5: 21–29. 23559820 10.4103/0975-7406.106559 PMC3612335

[70] McDowell MJ, Woffenden BJ (2003) Plant disease resistance genes: recent insights and potential applications. Trends Biotechnol 21: 178–183. 12679066 10.1016/S0167-7799(03)00053-2

[71] Thomine S, Wang R, Ward JM, Crawford NM, Schroeder JI (2000) Cadmium and iron transport by members of a plant metal transporter family in Arabidopsis with homology to Nramp genes. Proc Natl Acad Sci USA 97: 4991–4996. 10781110 10.1073/pnas.97.9.4991 PMC18345

[72] Williamson VM (1999) Plant nematode resistance genes. Curr Opin Plant Biol 2: 327–331. 10459002 10.1016/S1369-5266(99)80057-0

[73] Isokpehi RD, Simmons SS, Cohly HHP, Ekunwe SIN, Begonia GB, et al (2011) Identification of drought-responsive universal stress proteins in viridiplantae. Bioinform Biol Insights 5: 41–58. 21423406 10.4137/BBI.S6061 PMC3045048

[74] Yu H, Luscombe NM, Qian J, Gerstein M (2003) Genomic analysis of gene expression relationships in transcriptional regulatory networks. Trends Genet 19: 422–427. 12902159 10.1016/S0168-9525(03)00175-6

[75] Ferrell JE Jr (2002) Self-perpetuating states in signal transduction: positive feedback, double-negative feedback and bistability. Curr Opin Chem Biol 6: 140–148. 10.1016/s0955-0674(02)00314-9 11891111

[76] Zou M, Conzen SD (2005) A new dynamic Bayesian network (DBN) approach for identifying gene regulatory networks from time course microarray data. Bioinformatics 21: 71–79. 15308537 10.1093/bioinformatics/bth463

