# Correction: Identification of Differentially Expressed Genes in Leaf of *Reaumuria soongorica* under PEG-Induced Drought Stress by Digital Gene Expression Profiling

**DOI:** 10.1371/journal.pone.0104142

**Published:** 2014-07-28

**Authors:** The PLOS ONE Staff

There is an error in the grant number for the funding from State Key Development Program for Basic Research of China. Please refer to the correct funding statement here:

This work was financially supported by the State Key Development Program for Basic Research of China (973 Program, Grant No. 2013CB429904) and the National Natural Science Foundation of China (Grant Nos. 31070358, 30800122). The funders had no role in study design, data collection and analysis, decision to publish, or preparation of the manuscript.
