# Supplementary figures and images for: Identification of Differentially Expressed Genes in Leaf of Reaumuria soongorica under PEG-Induced Drought Stress by Digital Gene Expression Profiling
Source: PLoS One. 2014 Apr 15;9(4):e94277. doi: 10.1371/journal.pone.0094277 (PMC3988058; doi:10.1371/journal.pone.0094277)

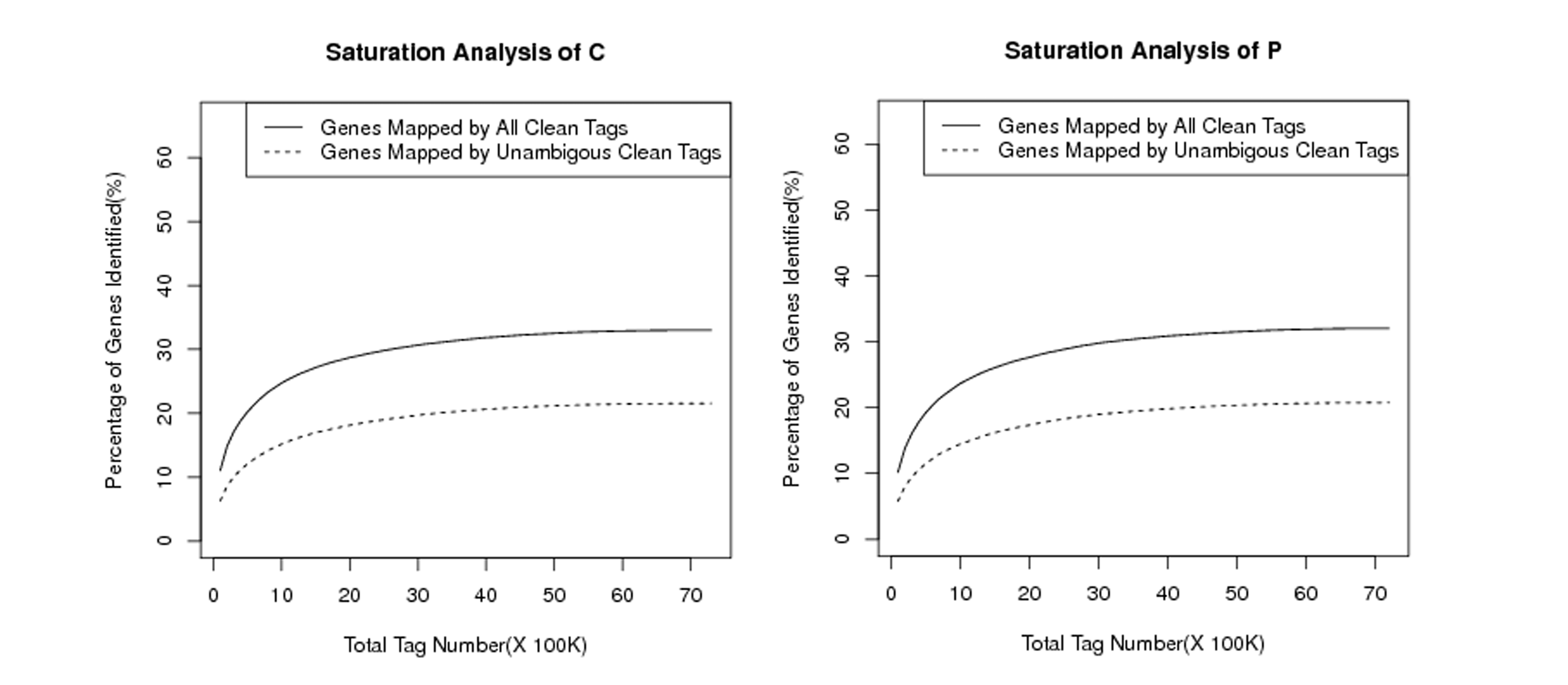

Supplement: Figure S1 — Sequencing saturation analysis of the two libraries. C represents the control group; P represents the PEG-treated group. (TIF) [file pone.0094277.s001.tif]

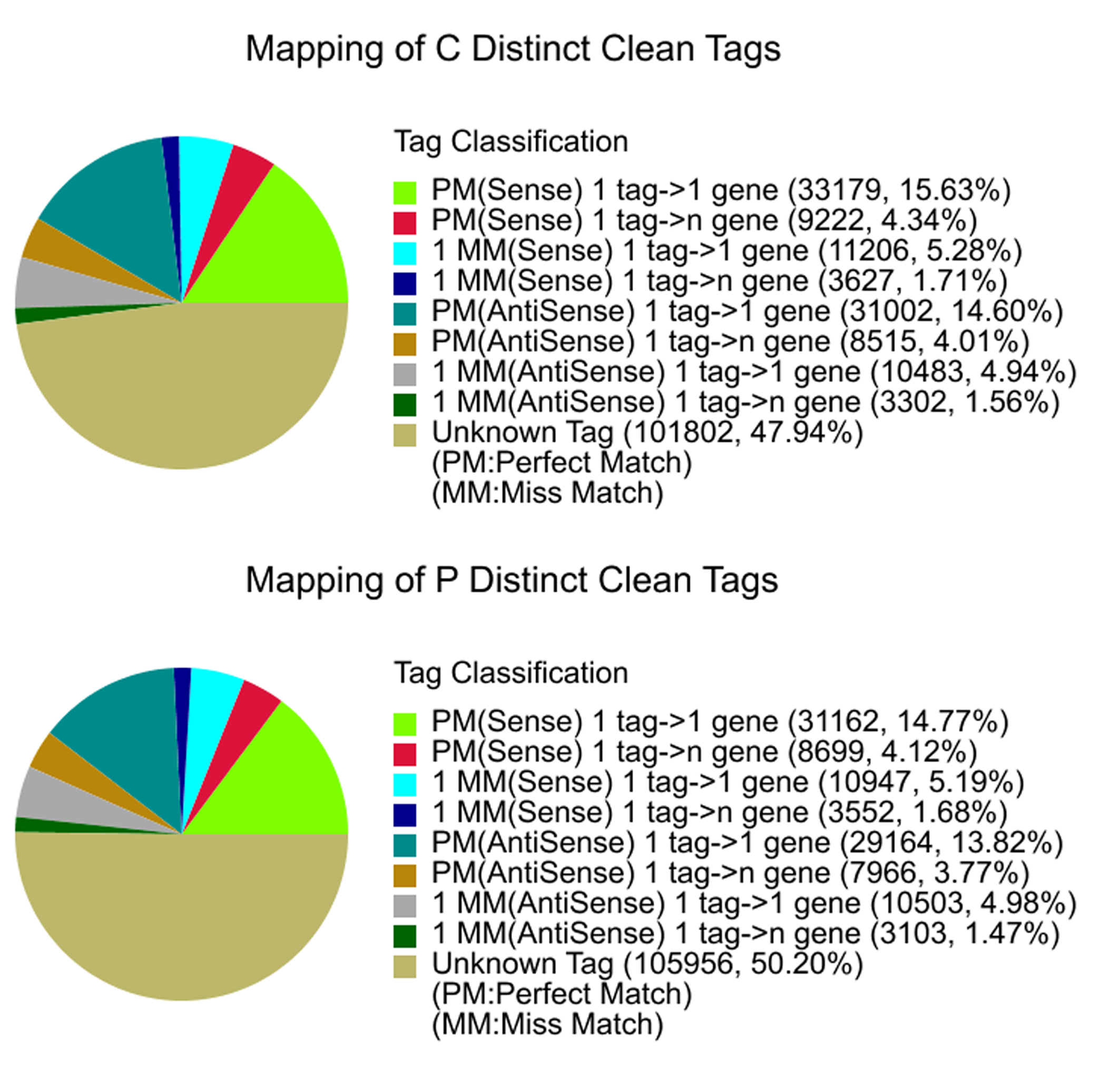

Supplement: Figure S2 — Mapping of distinct clean tags in the two DGE libraries. C represents the control group; P represents the PEG-treated group. (TIF) [file pone.0094277.s002.tif]
